# P12 Important early prognostic laboratory indicators for COVID-19 mortality

**DOI:** 10.1093/jacamr/dlac004.011

**Published:** 2022-02-16

**Authors:** Ergys Ramosaço, Entela Kolovani, Nevila Gjermeni, Najada Çomo

**Affiliations:** Faculty of Technical Medical Sciences, University of Medicine, Tirana, Albania; Infectious Diseases Service, University Hospital Center “Mother Teresa”, Tirana, Albania; Infectious Diseases Service, University Hospital Center “Mother Teresa”, Tirana, Albania; Infectious Diseases Service, University Hospital Center “Mother Teresa”, Tirana, Albania

## Abstract

**Background:**

COVID-19 has been marked as a highly pathogenic coronavirus of COVID-19 disease which has caused a pandemic into the human population, with a different course in different people, which contributed to the death of approximately over 5.02 million confirmed cases worldwide up till now. For this reason, it is quite important to have the earliest possible evaluation of laboratory-assisted indicators, especially for mortality risk prediction in developing countries, in addition to other clinical and imaging evaluations in order every physician to make the right medical decision.

**Methods:**

In total, 277 patients of Infectious Diseases Hospital, UHC “Mother Teresa” were enrolled. All of them tested positive for COVID-19. We evaluated indicators such as d-dimer (*N = *0–500 μg/L), lactate dehydrogenase LDH (*N = *95–200 U/L) and the neutrophil/lymphocyte ratio (control group = 1.7 ± 0.392) in two groups of patients, the first one of 144 patients who were discharged from the hospital and the second one of 133 patients who died.

**Results:**

What we observed was that the results were different for every test we performed in each group. Neutrophil/lymphocyte ratio was 8.69 ± 4.9 for the first group versus 14.7 ± 12.3 for the second one, *P<*0.05 (*P = *0.013), LDH = 427 ± 241 versus 715 ± 347, *P<*0.05 (*P = *0.0002), d-dimer = 1387 ± 763 versus 2650 ± 1322, *P>*0.05 (*P = *0.28).

**Conclusions:**

In addition to LDH as a prognostic factor, the neutrophil/lymphocyte ratio was considered as a low cost examination for severe prognosis and mortality risk (*P<*0.05), which can also serve as an alert for primary services for patient hospitalization, especially in developing countries after his clinical and image evaluation.

